# The Hospital. Nursing Section

**Published:** 1905-06-17

**Authors:** 


### The Hospital.

### Nursing Section.

Contributions for this Section of "The Hospital" should be addressed to the Editor, "The Hospital" Nursing Section, 28 & 29 Southampton Street, Strand, London, W.C.

No. 977.—Vol. XXXVIII. SATURDAY, JUNE 17, 1905.

### Notes on News from the Nursing World.

### THE NEEDS OF THE COLONIAL NURSING ASSOCIATION.

There were two conspicuous absentees from the annual meeting of the Colonial Nursing Association last week. One was Mrs. Francis Piggott, the originator and founder of the movement, who is abroad, and is therefore unable to take any active part in the work of the Association. The other was the Colonial Secretary. This is the second occasion of Mr. Lyttelton's absence from a gathering which, in the capacity of Mr. Chamberlain's successor, he has not yet attended. It was explained that his Parliamentary duties kept him from fulfilling his engagement to speak at Sunderland House; and, as a matter of fact, he addressed the House of Commons the same afternoon on the motion for the adjournment. But it is very unfortunate that he has so far been prevented from speaking on behalf of the Colonial Nursing Association. That organisation requires all the influence that can be brought to bear in its favour as well as all the financial help that can be secured. We rejoice that in respect to the latter, Sir Alfred Jones has set the splendid example of increasing his subscription of £50 to £250. The Association has now been in existence for nine years, and it certainly ought not to be hampered by lack of funds. Sir Alfred Jones rightly surmises that its aims and needs are not known so widely as they deserve to be.

### LADY WARWICK AND DISTRICT NURSING.

It is very important that the difference between ornamental and practical supporters of the nursing movement should be clearly defined. Without depreciating the worth of certain names, it is obviously the easiest thing in the world for people to allow their names to be used in the interests of a good cause. Unlike some notable personages, of both sexes, the Countess of Warwick does not consider that her obligations to the Warwick District Nursing Association begin and end with the appearance of her name as president. She makes a point of attending the meetings as often as she can, and of taking the chair at the annual gathering, instead of pleading a social engagement in London as an excuse for absence. At the annual meeting of the Association the other day, she delivered two admirable speeches, dilating in one upon the value of the work done, and in the other pleading for fresh support in order to avoid the unpleasant alternative of reducing the number of nurses employed in the town. That Lady Warwick's presence and her speeches were appreciated by the meeting was evident from the enthusiasm with which a vote of thanks to her was carried. Councillor Evans said that it was "a standing wonder how she found time to get through all her work, but she managed it somehow by industry and system." But where there is a will there is a way, and, generally speaking, the presidents of nursing organisations, whether women or men, whose assistance is limited to the use of their name and a small subscription, have little real sympathy for the cause.

### A POPULAR HOSPITAL MATRON.

The remarkable popularity of Miss Mary Hull, who for eighteen years served as matron of the Great Northern Central Hospital, and to whose work we alluded in announcing her resignation, has been attested in a striking manner. The reception for nurses, which we reported last week, and at which not only nurses new in training at the Great Northern Central, but nurses from all parts of England at one time associated with the hospital, were present, has been followed by a meeting of a more formal, though not more significant, character. Nothing, probably, could be more gratifying to Miss Hull than the numerous proofs which have been afforded her from time to time of the affection which is entertained for her by those who were trained under her, and are now scattered far and wide; while the speeches which were delivered at the interesting ceremony last week, and the presentations which were made on behalf of the committee, the medical staff, and the Ladies' Association must have given her the liveliest satisfaction as evidence of the high appreciation of her performance of her duties by everyone acquainted with her in the capacity of matron. Miss Pearse, her successor, who was trained at St. Bartholomew's Hospital and has lately acted as superintendent nurse at the North Stafford Infirmary, Stoke-on-Trent—where she had the pleasure of seeing one of the finest of modern Nurses' Homes opened on the crest of Hartshill—will have a stimulating example to encourage her, and we can wish her nothing better than that at the termination of her period of office, she may receive similar tokens of confidence and esteem.

### NURSING IN RHODESIA.

There is not much scope for private nursing in Rhodesia. Infectious cases are rare and all general cases, including malaria, go to the nearest hospital. Of these there are several, the "Memorial" at Bulawayo being about the largest. There are also several private mining hospitals situated on the mines. Salisbury boasts of a maternity home, under the auspices of the South African Extension Society, and Bulawayo has a private hospital for the same purpose For the ordinary private nurses there remains little save maternity work, and some of these cases are trying beyond description, particularly on the mines, in huts and tin houses, far away often from medical aid, with not much in the way of domestic help. Umtali, nestling among the hills, is very lovely, but extremely hot. The residents being for the greater part English, newly arrived from home, are very friendly and make the stay of an English nurse there pleasant.

### ALLEGED FRICTION AT SCULCOATES INFIRMARY.

Charges of "ragging" among nurses should not be lightly made, but if there be any apparent basis for them the authorities should lose no time in instituting a careful investigation. The rather vague assertions which were mentioned at the last meeting of the Sculcoates Guardians have been properly referred to the House Committee. The definite fact is that one of the nurses has run away and written to the master of the workhouse stating that the probationers in the infirmary have "a miserable time." This, however, may mean much or little, and as Dr. Lilley, at whose suggestion the matter has been referred to the House Committee, says that there is no material to work upon, it is doubtful whether the inquiry will justify the allegations to which several guardians have lent an attentive ear. We observe that during the discussion one of these gentlemen demurred to the idea that the probationers should take their orders from the superintendent nurse, and hinted that they should look to individual guardians for instructions. Any attempt to enforce this view might render discipline impossible, but the results would be very diverting to the onlooker.

### REJECTION OF A CURFEW-BELL RULE.

The House Committee of the Ashby-de-la-Zouch Guardians desire that the hour for the nurses returning to the house should be 9.30 p.m. summer and 9 p.m. in winter. A recommendation to this effect was discussed at the last meeting of the Guardians, and the Chairman of the Committee stated that, in his opinion, ten o'clock, the time at which the nurses had hitherto returned to the house, was a late hour for disturbing the patients. One of the Guardians said that it seemed to him that the proposal was like a curfew-bell rule, and that ten o'clock was not an unreasonable time for people capable of taking care of themselves. The recommendation was supported on the ground that discipline was essential, a resolution which was met with the rejoinder that as it was not alleged that the nurses had broken the rules, the question of discipline did not come in. On a division the recommendation was rejected by 18 votes to 13. The Guardians are therefore relieved of the risk of finding it extremely difficult to obtain nurses. Nurses are not children, and ten o'clock is a very reasonable limit.

### PARSIMONY AT KIDDERMINSTER.

The Kidderminster guardians have been discussing the question of the remuneration of probationers. At present they only pay their probationers £5 for the first year, and several of the guardians protest that the salary is inadequate. We agree with this view, and regret that the proposal to increase the salary to £10 a year was shelved. It was afterwards stated that an attempt to arrange that the probationers from the Kidderminster Union should finish their last year at Aston Union Infirmary in order to qualify them for a certificate which would enable them to take an appointment as a superintendent nurse, had not been successful. The reason, we gather, was that the Kidderminster guardians are not willing to assent to the financial conditions. It is essential to protect the pockets of the ratepayers, but parsimony, even from the ratepayers' standpoint, is often bad policy.

### LEEDS WORKING MEN AND DISTRICT NURSES.

At the quarterly meeting of the Leeds Workpeople's Hospital Fund Committee the Chairman referred to the establishment of the Maternity Hospital at Spring House, Caledonian Road, and to the proposal of the Leeds District Nurses' Committee to raise £10,000 for the purpose of enlarging their three homes. He affirmed that these are deserving objects, and the Committee would, he added, be asked to consider them when next they were allocating their grants. It is satisfactory to find that the labour of the District Nurses in Leeds is appreciated by the working men and their families in the town so warmly that substantial help is likely to be given to the scheme for the very necessary enlargement of the home.

### WORKHOUSE MATRON AS SUPERINTENDENT.

An excellent arrangement has been made at Elham Workhouse Infirmary. Miss Ralston, the superintendent nurse, having resigned her appointment, Mrs. Hillidge, who was recently elected matron of the workhouse, will henceforth discharge, in addition, the duties of superintendent nurse. The fact that Mrs. Hillidge is a trained nurse renders the conjunction of office not only suitable, but desirable.

### QUEEN'S NURSES' NEEDLEWORK GUILD AT NORTHAMPTON.

The fourth annual sale of work at the Victoria Nurses' Home, Northampton, was opened on Saturday by Earl Spencer. The lady superintendent, Miss A. F. Lunn, and the nurses under her, who form the Queen's Nurses' Needlework Guild, of which Princess Christian is patroness, entered upon their task with vigour, and the result was a small but admirably arranged number of heavily-laden stalls, set out in a tent on the lawn of the institution. A specially pleasing feature was the interest taken in the sale by poor patients who sent in many small contributions. The nurses' sitting-room was nicely arranged for concerts, and refreshments were served in the dining-hall. Lord Spencer, in performing the opening ceremony, having expressed the regret which he was sure they would all feel at the resignation, through ill-health, of Mr. John Cooper, from the chairmanship of the committee, said that the institution was doing a splendid work and deserved every help and support. Proposing a vote of thanks to Lord Spencer, Mr. F. G. Admitt intimated that the governors of the Northampton General Hospital were thinking of reviving a nursing adjunct, but he trusted that it would in no way be a rival to that institution. He hoped that there would be a mutual understanding whereby the two institutions could be run on harmonious lines, especially as at the present time many of the out-patients of the hospital were attended by the Queen's nurses. We hope that the sum realised by the sale of work is a substantial addition to the funds at the disposal of the managers of the Nursing Institution.

### NEWARK AND THE QUESTION OF FEES.

The movement for the formation of a nursing association at Newark has been taken up with an eagerness which augurs well for the future. The mayor of the town presided at the inaugural meeting, and after it had been explained that the nurse already employed by the St. Leonard's Charity was fully occupied, it was decided to form an organisation and to affiliate it to Queen Victoria's Jubilee Institute. The cost, it was stated, would be from £85 to £100 per annum. Subsequently, it was proposed that the services of the nurse should be restricted to members who make a small monthly or annual payment, and to non-members who pay on a higher scale during illness, and the proposal was agreed to. But we hope that this will not be a hard and fast rule. It is of the essence of the principles of the charity founded by Queen Victoria that the nurses employed under its auspices should be allowed, if necessity arises, to attend the sick poor free of all cost.

### THE TRAINING AT SHOREDITCH INFIRMARY.

The marriage of Miss A. O. Appleyard to Dr. Victor Bonney is an event of interest to many former nurses at Shoreditch Union Infirmary and other institutions. Miss Appleyard, who was trained at St. Bartholomew's Hospital, and after served as sister at the Chelsea Hospital for Women, became assistant matron at Shoreditch Infirmary, and had charge, in the absence of a trained matron at the latter institution, of the training and nursing arrangements, resigning her appointment when Miss Atkins was elected matron. We are informed that during the period of her association with the infirmary, and prior to the appointment of Miss Atkins, the first trained matron, certificates were gained by a number of nurses, and that those who worked under Miss Appleyard greatly appreciated her sympathy and her efforts to raise the standard. Her last appointment before her retirement from nursing was that of sister-in-charge of the St. George's Hospital Nurses' Home.

### GRATUITIES TO CANTERBURY NURSES.

It is one of the rules of the Kent and Canterbury Nurses' Institute that every nurse who has served satisfactorily for 12 years shall receive a gratuity of £10. The Committee always take the opportunity of having a pleasant social meeting on the occasion of these presentations. This year two nurses, namely, Miss Juliana Brockett and Miss Alice Gearing were qualified to receive this gratuity, and it was hoped that it would be possible to have the usual garden party in the pretty garden of the Institute on Tuesday, June 6, but the heavy rain made it necessary to hold the reception in the rooms of the Institute house. Nearly 50 of the friends of the Institute assembled, under the presidency of the Bishop of Dover. Mrs. Mapleton Chapman presented the two nurses and asked the Bishop to hand to them their cheques and illuminated certificates. In Miss Brockett's case a grateful patient had added a gift of £2. The Bishop's address was much appreciated. Mr. Chapman returned thanks on behalf of the two nurses. The company were then entertained at tea, and the nurses received the congratulations of their friends.

### ANOTHER NEW CLUB.

There has just been opened another new club which it is thought may prove attractive to nurses who, having private means, can afford to join it. Situated at the corner of Holland Park, the London County Club is founded principally for the association in membership of ladies engaged in professional or commercial pursuits. There are between 30 and 40 bedrooms—some for two friends—which can be furnished by the occupants if desired. The two dining-rooms will accommodate 70 persons, and the several sitting-rooms are in excellent taste. A feature of the club is the employment of ladies as the domestic staff, the "waiting maids" being prettily and quaintly attired. It does not, however, appear that anyone of prominence in the nursing world is connected with the movement.

### A HOME FOR WEST HARTLEPOOL.

At the annual meeting of the West Hartlepool District Nursing Association, the Mayor moved the adoption of the report, which shows that the number of patients visited was 370, and the number of visits paid nearly 11,000, a considerable increase on the previous year. The income was £795, but this included a balance of £416, and since March 31, the sum of £300 has been invested in Corporation bonds. Therefore, as the Mayor pointed out, it is necessary to spare no exertion to secure a sufficient number of subscribers. Subsequently, it was suggested as a remedy for constant changes in the nursing staff that a comfortable house should be provided for them, and as the association possesses invested funds, we think that the suggestion should be considered. The organisation has now been established 15 years, and as the work of the nurses is still growing, the time seems ripe for making the attempt to afford them the suitable accommodation which at present is lacking.

### SHORT ITEMS.

Miss Helena Hartigan, Miss Ethel Maud Rentzsch, and Miss Margaret Bessie Williams have been appointed staff nurses, provisionally, in Queen Alexandra's Imperial Military Nursing Service. Miss I. M. Robinson has been confirmed in her appointment as staff nurse.—As usual at Whitsuntide, the chapel at St. Mary's Hospital was tastefully decorated under the superintendence of the night sister, the home sister being responsible for the musical arrangements. There were three celebrations of the Holy Communion on Whit Monday; one at 9 a.m. specially for the night nurses.—The Secretary of the Maternity, Charity, and District Nurses' Home at Plaistow inform us that the number of midwives trained at that institution last year was 1,230 and the number of nurses 445.

### The Nursing Outlook.

"From magnanimity, all fear above;
From nobler recompense, above applause,
Which owes to man's short outlook all its charm."

### ECONOMY IN HOSPITAL WORK.

THERE is nothing which is more required in the present day in the best interests of sound hospital administration than the enforcement of economy in every department of the hospital. By economy we mean the management, regulation, and supervision of every article in use. Wise teachers have always enforced the principle that economy no more means saving than it means spending money. It means the use of money, or time, or material, or anything, to the best possible advantage. A hospital possesses many, more than the usual, impediments to economy, owing to the varied composition of the household. Each department is necessarily to a greater or less extent under distinct management, and in most hospitals, especially if there be no medical superintendent, the controlling hand over every detail of the daily work, for effective purposes, may be absent.

The essential of successful control in large establishments is the existence of a spirit of responsibility which permeates the whole organisation and forms an active principle in the character of every responsible person within its walls. Each medical officer is not only responsible to the institution for the patients' welfare, but it is also his duty to provide, that in all the arrangements in his wards, and in all the details of treatment and dressing, waste shall be avoided, and economy enforced.

The lady superintendent is responsible for the character and principles on which the probationers and nurses discharge their duties, for their welfare and comfort, and she has also to see that none of her staff are wasteful in the use of hospital stores. The sister is responsible for the well-being and order of her ward, its discipline and arrangements, including the comfort of both nurses and patients. She has also, through the lady superintendent, to take care that each person serving under her is impressed with a feeling of responsibility, and that she exhibits it in the use of hospital material of every kind. In practice, there is reason to fear, however, that the lavish expenditure now in fashion in many great hospitals, which finds expression in elaborate and expensive buildings, in equipment of the most costly kind conceivable—which sometimes, as in the case of many modern operation theatres, may be quite unnecessary—has a tendency to make the humbler workers careless in the use of stores and dressings, with results which materially affect the cost per patient and per bed each year. Nevertheless, as the quality and character of the workers in the nursing department have improved, a better spirit and a keener sense of responsibility has undoubtedly arisen.

We believe that the real weakness in control is due in the main, so far as the nursing staff is concerned, to imperfect educational methods. It has been well said that a nurse who has had no lessons in economy at home, and who comes to the hospital without any training in this direction, will never make an efficient administrator. Be this as it may, it is an undoubted fact that facilities are needed in this country for the instruction of intending probationers in what are known as hospital economics These should include both method and practice; careful instruction in the use and purposes of all dressings and appliances employed in the treatment of disease, with a knowledge of their relative values and cost; the production and manufacture of food, its preparation for the table, and its economical purchase and use; the handling of stores; the importance of bacteriology and physiology in relation to food, to diet, and disease. If each nurse had a good grounding in dietetics and household economics she would strive to prove herself the handmaiden of economy, rather than an irresponsible waster of hospital stores. The prevention of waste is a duty which should be enforced as a matter of education upon each member of the hospital staff. At present, unfortunately, so many are ignorant of the cost of equipment and supplies, as well as indifferent to the exercise of the true economic spirit, that the consequences when expressed in waste and cost are indeed serious.

This question of economy is one which affects the character of all who are engaged in the work of a hospital. We believe that the speediest way to the application of an effective remedy would be for each hospital to appoint a small committee, consisting of one honorary physician, one honorary surgeon, the matron, the superintendent or secretary, and a representative of the sisters and of the staff nurses. They should be instructed to inquire into the working and arrangements of each ward and operation-theatre in regard to the use of every article of consumption, with the view of recommending what steps it is desirable to take in order to enforce uniformity, as far as practicable, in the use of all materials, everywhere, throughout the establishment. If this step were taken, and if the authorities were to consider how they can best provide for instruction in hospital economics and dietetics, within a few years it is certain that large reductions in expenditure might be found possible, in many hospitals, where too little attention is given to these matters at the present time. A recent inquiry revealed, that in one hospital a saving of £40 in one year could be effected in safety-pins alone, and on further investigation it was discovered, that some hospitals of equal size spent less than £40 on this one item, each year. It is not fair to blame the nurses for wastefulness in any hospital, where due regard is not paid, to the enforcement of a spirit of responsibility in respect to the use of hospital stores, of all kinds, on all occasions. No doubt it is very difficult to keep a tight hand upon every item, and to see that waste is checked from day to day. Still the cost of not enforcing this control, and of not educating the staff to their responsibilities, in the use and abuse of hospital stores, has become so costly a matter, in these days, that every well-regulated hospital owes it to its reputation, to face the difficulty fairly and squarely, and to promptly apply an effectual remedy.

### The Nursing of Sick Children.

By James Burnet, M.A., M.B., M.R.C.P.Edin., Registrar, Royal Hospital for Sick Children; Clinical Tutor, Extramural Wards, Royal Infirmary; and Physician to the Marshall Street Dispensary, Edinburgh.

### XI.—RICKETS AND SCORBUTUS.

In this lecture we shall consider two diseases, one of which is of very common occurrence, while the other is much less frequently met with. The disease known as rickets is very apt to suggest to the nurse's mind a condition marked by deformity of the bones, and so it is to a certain extent; but the bending and other changes which take place in the bones during the course of this disease are not the only symptoms. Rickets, in fact, is more a disorder of nutrition than an affection of the bones. Accordingly it will not be surprising to learn that other symptoms present themselves for consideration in children who are the subjects of rickets. One of the most frequent of these symptoms is restlessness, which is very marked during sleep, the child throwing the bedclothes off. In this way a chill is often contracted which leads to bronchitis, or even to pneumonia. The nurse must, therefore, attend to this symptom, and endeavour as far as possible to prevent the clothing from being thrown off. The child should wear a pyjama sleeping suit, and the bedclothes should be warm but not too heavy.

Sweating is another common evidence of rickets. This sweating occurs chiefly about the head, so that the pillow is wet in the morning. A third symptom is tenderness, the patient crying, or even screaming loudly whenever he is lifted or moved about as in bathing. The nurse should bear this symptom carefully in mind, and endeavour to move the child as little as possible. In addition to these symptoms we have a condition of general malnutrition, sometimes accompanied by diarrhœa and vomiting. Such children are often found to be very pale and anæmic-looking. They are late in cutting their teeth and also in walking. The opening in the skull is apt to be long in closing, while the head sometimes tends to assume peculiar shapes, one of which is specially characteristic and is known as the bossy skull. The skull in this variety presents two well-marked prominences or bosses over the forehead. The ribs are often found to be thickened at a point some inches from the breastbone, and these bony knots are so like beads to the touch that they constitute what is familiarly known as the rickety rosary.

So far we have considered those symptoms of rickets which are more or less common to every case of this disease. We shall now look at one or two which are not always present, but which, when they do exist, constitute special conditions calling for very definite treatment. The first of these we shall refer to is bronchial catarrh. This is very common in rickety babies. It may be induced by the patient throwing off the bedclothes during sleep. It is not in itself a dangerous condition, but it may lead eventually to severe bronchitis or even to pneumonia. The nurse must accordingly attend to bronchial catarrh whenever it occurs. The baby should be kept warm, but must on no account be coddled up in a close atmosphere with a superabundance of shawls and other wraps. The application of a little turpentine liniment to the chest at bedtime is useful, but as a rule cough mixtures do more harm than good.

The occurrence of convulsions is specially common during the course of rickets. They are often induced by constipation, or it may be by the cutting of a tooth. Most frequently, however, some disturbance of the digestive tract is the source of the convulsive seizure, and the nurse must, therefore, be careful to avoid giving anything to rickety children which is at all likely to interfere with digestion. When a fit occurs cold should be applied to the head and warmth to the feet. The use of the hot bath is not always advisable in these cases. When the fit is over a dose of castor oil and special care in feeding will be necessary. A condition known as tetany is sometimes met with. In this we find spasmodic contraction of the upper and lower limbs. This contraction is apt to be more or less prolonged and may be associated with a considerable amount of pain. The nurse in such cases may try the effect of hot baths, combined with gentle massage of the affected limbs. Douching of the spine often has a very beneficial effect.

Sometimes spasm of the larynx may occur. In this condition the child has an attack of crowing, in which the breath is held for some seconds and he appears as if being suffocated. Relief comes when the child gives a prolonged crowing sound and the attack is then over for the time being. These attacks are apt to be very alarming. They may be induced by fright or by a draught of cold air. In some instances the patient wakes out of sleep and forthwith has a severe laryngeal spasm. In order to afford relief a sponge filled with hot water should be applied over the throat. Afterwards the case should be treated by daily cold spinal douching and strict attention paid to the general rules which are given below for the treatment of rickets.

Regarding deformities of the limbs a few remarks must suffice. The deformities include bending both of arms and of legs, the latter being most frequently affected. In bad cases the patient must be absolutely prevented from walking. To accomplish this object splints, previously padded, should be applied to the limbs reaching from the lower part of the chest to beyond the heels. These splints should be put on somewhat loosely in order that the circulation may not be at all interfered with and growth thereby impeded. In milder cases massage and douching will suffice.

The following rules will be found useful as guides to the nurse in managing cases of rickets:—

1. Attend to the diet. Feed the patient regularly. Milk should form the main constituent. After the first year see that the child has in addition well boiled oat flour, eggs, and gravy. Fat in any form is to be commended. Avoid all artificial foods.
2. See that the child has abundance of fresh air and sunlight.
3. Do not overclothe the patient, but see that the abdomen and limbs are thoroughly protected.
4. Give cod-liver oil in the form of Nutrol, or use

Virol, both of which are rich in fat, and should be regarded as foods rather than as medicines.

We must now pass on to look for a little at scorbutus, or as it is perhaps more familiarly termed, infantile scurvy. This is a disease produced by feeding infants on artificial foods or on milk which has been sterilised. The leading symptom of this disease is tenderness of the limbs, so that the patient screams whenever any attempt is made to lift him. After a time the limbs become swollen. In addition, we may have hæmorrhages of all kinds. Thus bleeding from the bowel is not uncommon; and if the patient has cut any teeth the gums may be very soft, spongy, and found to bleed very readily.

The measures to be adopted in the management of these cases are simple enough. In the first place the nurse must attend to the feeding of her patient with very great care. All patent foods must be stopped immediately. The milk must no longer be boiled, but must be given fresh to the infant. In addition a teaspoonful of lemon or of orange juice should be given to the patient two or three times a day, while raw meat juice may often be employed with advantage. Great care must be taken in handling the child as spontaneous fracture sometimes occurs, and the nurse is apt to be blamed in such cases. As the limbs are very tender pressure of the bedclothes should be avoided by placing cages, such as are employed in fracture cases, over the lower part of the body. Attention must also be paid to fresh air and sunlight which are of considerable importance in the treatment of scorbutus as well as in that of rickets.

### The Nurses' Clinic.

### NURSING AND FEEDING OF CASES OF ACUTE DIARRHŒA AND VOMITING.

As the hot season is drawing near, a few words on nursing and feeding infants suffering from the much dreaded summer plague, diarrhœa and vomiting, may be of service to my fellow nurses.

We all know the aspect of these poor babies—the anxious expression, the restlessness, the hollow eyes, the frequent piercing shriek, the shrunken abdomen and drawn-up legs.

As a rule the temperature is subnormal and the baby collapsed on admission, probably the result not only of the disease itself but of the frantic efforts of the mother by trying every conceivable artificial food and also every soothing "powder" to overcome her darling's condition.

In any case a nice hot bath will prove beneficial It soothes the child, subdues the pain, allays to some extent the thirst. If the baby is very collapsed, the addition of some mustard to the bath will be a help.

Then put the child to bed, dressed lightly and warmly, give a hot bottle, and apply hot flannels or hot wool to the abdomen; clean out the mouth well with a mild mouth-wash or cold water only, and then try feeding. If the sickness has been very troublesome, it is best to start straight away by giving albumen mixture, composed of sterile normal saline solution O j and the whites of two eggs well beaten up. If the child objects very much to the taste, a little sugar of milk or ordinary loaf sugar may be added; but, as a rule, these babies are so fearfully dried out and thirsty that they will drink anything, and it is piteous to see them start and look round with a hungry, anxious expression when the glass is touched with the spoon. If the child keeps a drachm or two of this mixture down, increase the dose gradually, and if the sickness does not return, let it drink as much as it likes after a few hours. The great thing is to make up for the enormous loss of fluid and the more it will drink of this good mixture the better.

If there is still inclination to retching give small quantities frequently, about a drachm every 10 minutes or less. If even this causes sickness the doctors generally stop all feeding by mouth and order normal saline solution to be injected subcutaneously. The apparatus required is an ordinary brass syringe, a piece of indiarubber tubing and a small exploring needle, all well sterilised. The sites for injection are those where the skin is easily lifted off and where the underlying tissues can readily accommodate the fluid. There is generally no difficulty in getting the fluid absorbed in these emaciated babies. The subaxillary and intracapular spaces, and the groins are the most usually chosen, the skin is well purified in the ordinary way, and the fluid injected very slowly (five minutes to each ounce), having carefully excluded the air from the syringe before inserting the needle. The temperature of the normal saline solution is about 104° Fahr. As a rule not more than four ounces are injected at one operation, and the ordinary brass syringe just holds this amount; should it, however, be necessary to refill the syringe, be very careful to stop the lumen of the indiarubber tube by a pair of Spencer Well's forceps before taking off the syringe, and again carefully exclude any air-bubbles before refixing the syringe to the tube. A small collodion dressing is applied when the infusion is completed. Babies are often fed in this artificial manner six times hourly for 24 hours—which will give their stomach a complete rest—and then feeding by mouth with albumen mixture is started again very gradually, reducing the subcutaneous feeding correspondingly, and ultimately leaving it off altogether. The mouth wants constant attention, and children who are very sick will only stand cold water, anything with a taste starting the vomiting again—the more they swallow of the water the better—they like the fresh feeling, and will suck the wet rag with avidity. When the babies have got to the stage of taking the albumen mixture without showing any symptoms of returning it, one can try adding milk. This is done in the form of either peptonised milk or Benger's food; a drachm of any of these is added to the ounce of albumen mixture, and if borne well the strength increased until the patient is able to take either weak peptonised milk or Benger's food alone. Before returning the child to the mother an effort must be made to get it back on ordinary milk. There is generally no difficulty about this; the milk will have to be well diluted at first. Lime water and sugar of milk are very excellent for this purpose, and I have found a special preparation of oatmeal—"Knorr's Hafermehl"—a most successful dilutant and very popular with the small patients.

The babies are best nursed in curtained cots, and thus kept absolutely quiet. It has been a frequent experience with me to observe that babies who after having had their feed had their curtain drawn at once and were kept in absolute "retreat" have overcome their sickness quicker than those left in open cots. It is wisest to clean the mouth before feeding during the acute stage, as any irritation or excitement after food may cause it to regurgitate.

Frequent baths are must useful. They must be given at least twice a day; any method of putting the dried-out patient in contact with water is beneficial. In addition to the care of the mouth, the greatest cleanliness must be

### THE NURSES' CLINIC—*Continued.*

observed with regard to the excreta. The fæces must be considered as infectious, and the diapers and nurses' hands treated correspondingly. A great deal can be done in this way to prevent the spread of the complaint. In some hospitals in Germany so much stress is laid on this point that special nurses are told off for the feeding, and special nurses for the cleaning-up of the children suffering from acute diarrhœa and vomiting. This luxury is not possible in most English hospitals, but with conscientious attention to scrupulous cleanliness the results can be satisfactory.

Meat juices in small doses are added to the albumen mixture by some doctors, and do a certain amount of good as stimulants if kept down. Brandy or other forms of alcohol by mouth seem to increase the tendency to sickness in infants, and if found necessary are best given hypodermically some time before food, so as to get the baby to be in a quiet frame of mind for feeding.

Drugs do not come into the province of the nurse, and few are used with success in this complaint. An initial small dose of castor oil, before starting feeding, or repeated doses of an alkaline mixture of ol. Ricini have occasionally seemed to prove helpful.

### Select Committee of the House of Commons on Nursing.

### THE SCHEME FOR TRAINING-SCHOOL REGISTRATION ELABORATED.

#### Evidence of Lady Helen Munro-Fergusson.

Lady Helen Munro-Fergusson was the first witness examined by the House of Commons Select Committee on Registration of Nurses on Tuesday, the 6th inst. In her opinion the present state of things in the nursing world was not very satisfactory. The certificates of the various hospitals varied very much in value and the public was unable to distinguish between their merits. They varied both in training and in examination, and it would certainly be to the advantage of the public that this variation should cease and that there should be some sort of co-ordination. The certificate of a Central Board would be evidence that a nurse had been duly trained under satisfactory conditions and had received a good character from her matron. In her experience it was difficult to get records at present, and in case of illness there was no time to make inquiries as could be done with a teacher or a domestic servant. In her opinion, too, the average matron would hardly have time to carry out her ordinary duties and also those of a registry office. Under a system of State registration the Central Board might make inquiries as to character and efficiency. The effect on the hospitals would be to level them up to the standard set by the Central Board, to the great advantage of the hospitals and of the country. The principle of examinations was already accepted; the only difference under State registration would be that they would be conducted independently and with a uniform standard. The Central Board of experts must define the standard. She thought that doctors and employers would report cases of misconduct, and the nurses would be taken off the register. The registration of Nursing Homes could be done by the local authorities, but that would not meet the case of registration of nurses. They would still have to have an expert committee to define the standard and see that it was adhered to. There should certainly be a standard to which all hospitals throughout the country should work, and so all would turn out properly qualified nurses. They wanted a curriculum laid down by an expert body so as to get a minimum of uniformity.

#### Evidence of Sir Henry Burdett.

Sir Henry Burdett was then examined upon the evidence he gave on the 25th ultimo and upon the scheme he then submitted for the Committee's consideration.

With regard to the course of training for nurses, taking the 140 institutions with 100 beds and upwards, he found that of the 38 in London, 37 had a three-years' course, while the remaining one had a two-years' course and retained the services of its nurses for another two years, rising them as staff or private nurses during the third and fourth year; but that was employing them on the work of the hospital rather than on their education. In the provinces and Wales, of 78 hospitals, 76 had a three-years' course, and in the remaining two the course was two or three years. Of the 14 in Scotland, 13 had a three-year course and one two- or three-years'; of the 10 in Ireland, nine had three-years', and one one or more. Of the 80 institutions with under 100 and not less than 50 beds, in London all six had three-years'; of the 61 in the provinces and Wales, 51 had three-years', four two-years', three one-year, one four-years', and two two- or three- years'. In Scotland, of five, three had three-years', one two-years', and one two- or three-years'; and in Ireland, of eight, five had three-years', one four-years', one four-and-a-half-years', and one three- to five-years'. Summing up, it might be taken that the three-years' course had been established; and he would like to repeat that this had been secured by the resolute persistence of Mrs. Bedford Fenwick. The few exceptions would, he had no doubt, in practice soon level themselves up to that standard. As to the quality of the training given, that was quite another matter.

Replying to questions by the Chairman (Mr. Tennant) in review of his evidence generally was of opinion that, clearly something must be done; and what he advocated was the registration of all nurse-training schools and of all agencies which supply nurses to the public. The sources of supply must be absolutely controlled, and if that were so the pseudo-nurse must die out; she would never get employment. Only those should be recognised who attained a certain standard of training and efficiency, and the certificate given should have a definite and recognised value. Under his scheme the training schools would have a definite standard of education and would certify that that was so.

In order that they should all attain a recognised standard of efficiency it was essential that the General Nursing Council to be set up should supervise the examination of all nurses. This council should represent the brains—the leading people—in nursing education, in the same way that the General Medical Council did in medical education. He attached great importance to uniformity of curriculum; mere visitation without supervision of examinations would not secure this. The Nursing Council, he suggested, should have authority to supervise the examination papers and so see that the nurse who got a certificate had attained a minimum standard of efficiency. What would happen then would be that the Council would discuss with the schools the class of question to be set. There must be some practical form of control, since it was very difficult to get anything like uniformity with voluntary institutions. There was no doubt whatever about the general feeling as to a desire for improvement; and if the General Council consisted of all that was best in connection with the training of nurses, it would bring about co-operation between the various bodies, and so set up a common standard.

### SELECT COMMITTEE OF THE HOUSE OF COMMONS ON NURSING—*Continued.*

He did not suggest the setting up of an autocracy, to rule with a rod of iron, but of a representative body which would carry into effect the views of the nursing world. By supervision he meant inspection and control—the pointing out, for instance, that certain questions were undesirable; that the standard of knowledge required was too high, or too low, and so forth. The system he suggested would bring all nurse-training schools into line at once, because to be able to train they must be registered, while under the Bills now before Parliament it would be inoperative, and many of the nurse-training schools were absolutely opposed to it. St. Thomas's, King's College, the Middlesex, the Royal Free, Guy's, Sick Children, Charing Cross, London, St. Mary's, University College, Westminster, and the National for the Paralysed, had all declared against the system of registration as set forth in these Bills, the last six named having passed specific resolutions against it. So far as was known only about 1,500 nurses were waiting to register. The Incorporated Society had been working most vigorously and energetically; they had published the names of everyone who had joined and that he believed was the number. The Royal British Nurses' Association had a system of voluntary registration, and from first to last 2,500 was the extent of it, trained or not. He maintained emphatically that if they passed an Act for registration of nurses it would not accomplish what they were striving after. They ought to rest on the bed-rock of the feeling of the country in this matter and he believed that the great majority of the nurses of the country desired some scheme which would protect the capable woman. And it was because he sympathised with this as a matter of national necessity that he wanted the Committee to set out a plan which would draw together all those engaged in training. This he thought would be attained by the registration of the training schools on his plan.

The Chairman: Do you consider that those who are opposed to registration as in the Bills will agree to that?

Sir Henry believed that if they were brought in and recognised as they would be by being represented on the Council the plan would be welcomed by the great body of nurse-training schools. No doubt, of course, some would oppose it; but they could not sit still because some institutions had peculiar views. Such an attitude was hopeless for progress but it existed and always had existed. What they had to do was, in the least objectionable way, to bring the majority to co-operate together. They must have some representative authority which would have control over the training schools, but that control must be voluntarily accepted. They could not carry reforms unless they carried the institutions with them. He certainly believed that the great majority would accept some such scheme.

"You would not be afraid," asked the Chairman, "that some who might stand out would be producing better nurses?"

"No," said the Witness, "if the registration of training schools be enacted by Parliament they will all register for the simple reason that no school could exist which did not. Nurses would not go there to be trained if the school could not give them the necessary certificate. After all nurses are working women and have to get their living, and they would not waste their time in that way: a non-registered school could not go on."

"Do you suggest that the Nursing Council should have the power of entry into hospitals to see if the prescribed curriculum was being carried out?"—"Not exactly; the Council would lay down the standard of efficiency and the schools would accept it, but there would be no need to harrass them. The Council would have the right of supervising the examinations and of visiting. I do not think the hospitals would object to that. They are visited by the King's Fund and no objection is raised. I myself have been denounced as a critic; I have been a critic for 40 years, but there is no institution that does not welcome my visits. I feel sure the hospitals would welcome registration if they were properly represented on the Council and so had a voice in the management of affairs. Certainly there might be some difficulties at first. Whatever is suggested there may be difficulties, but there is a spirit abroad in favour of improvement and levelling up and we have every confidence that no real difficulty will be experienced. If the scheme is in the interests of the nurses, of the medical profession, and of the sick, the idiosyncrasies of individuals must not be allowed to weigh against it. After all, government in this free country is by consent and the people concerned are behind the demand for an alteration of the present system."

Questioned by Mr. Pierpoint, Sir Henry said the thing to aim at was uniformity of examination by having the same papers all over the country. They must begin with a Nursing Council, make it as representative as they could, and then leave it to decide how far it could go. Probably at first they would not get uniformity. The visitors would point out defects and differences and progress would be gradual. No drastic or Napoleonic scheme was required, but they must legislate along the lines of carrying the schools with them by friendly co-operation. Certificates would be given by the individual schools, and they would secure the seal of the Nursing Council just as the diploma of the medical man secured that of the Medical Council. The examining bodies would set forth that certain requirements had been fulfilled, and the nurse would be registered in consequence.

Would you prevent the common woman—the neighbour, say—from nursing in an emergency, even if paid for her services?—Certainly not; there is always a clause providing for that effect in all Registration Acts.

By Dr. Hutchinson: Witness quite agreed that nursing had vastly improved during the past 20 years, and especially in the last five, but that now something more must be done. The proposal for State registration of every nurse he did not think practicable, and his alternative was that, instead of the individual, they should first register the schools. Then they could lay down certain rules—that they must have a standard of education and examination; be licensed to carry out examinations; and as soon as the nurse got her certificate she would automatically go on to the register. A General Nursing Council would be formed to supervise the examinations and training, and have charge of the register, and the registration would be without expense to the nurse. Then if a nurse were not on the register she would practise at her own risk, the same as a quack doctor, and the persons who took her would take her at their own risk, so long as she did not give herself out as registered. The registration of the schools would imply uniformity of standard. Then also all homes and institutions that send out nurses to employers should be registered, but that should be by the local authority not by the Nursing Council. Education was one thing, supply another. So also with massage establishments, just as was being done with registry offices for servants and other employment agencies.

Rural nursing was a separate matter. It had not had so long a run, was in fact in its infancy. It was only established some ten years ago, and especially during the past five years it had shown great vigour, and was doing an enormous lot of good. It was important that it should not be interfered with by legislation in any way, but it should be possible for the General Nursing Council, after a few years, to turn its attention to the schools for training village nurses into

SELECT COMMITTEE OF THE HOUSE OF COMMONS ON NURSING—*Continued.*

cottage hospitals, and small institutions, but at present these should be left outside.

In reply to Mr. Hobhouse, Sir Henry reaffirmed his statement that all training schools worthy the name did in fact keep registers of their own. Then as to the composition of the suggested Nursing Council, he said it would be made up of trustees of training schools, teachers and controllers of nurses, matrons, and doctors, and also of direct representatives of the nurses themselves, as was the case with doctors under the Medical Act. If they followed that Act there would be five nurses as elected representatives—three for England and one each for Scotland and Ireland. The larger proportion of members of Council would probably be those directly engaged in the training and education of nurses, who could serve in turn. The general public would be amply represented by the lay element of the trustees of hospitals, since the matter was a highly technical one and what the public wanted was the higher and better education of nurses.

Sir John Stirling-Maxwell asked how many training schools might be expected to register? to which the reply was from 200 to 250. There were 220 institutions with 50 beds and upwards training nurses at the present time. It might be more, since under the Nursing Council the whole body would probably be gathered up in some affiliation scheme by which a complete course of training could be afforded to every nurse. It was at present a fact that no hospital in the world could give a complete curriculum in every branch of nursing. He had no doubt as to a Nursing Council being able to manage as many as 200 or 250 schools. They would be organised by counties probably, and he thought they might look for an advance in management generally.

To Major Kenneth Balfour Sir Henry replied, on the question of nurses going back to their schools in order to keep efficient by post-graduate classes, that though there might be increased outlay necessitated for the increased accommodation required yet there would be increased revenue also, reminding the Committee that Mr. Holland said that the London Hospital made £4,000 a year by its private staff. Undoubtedly the profits to be made were large.

Questioned by Mr. Mount and the Chairman regarding the supply of nurses, the witness expressed the opinion that there would be no real difficulty in getting all the nurses that would be required. As to existing nurses, he thought they certainly should be given a chance to show what was in them, and by a system of examinations by the Incorporated Society to give them certificates and so allow them to register, for generally speaking they were a most excellent class of woman. There must necessarily be an interregnum before a new system could be in full working order. The Society might do much good in these two years. His final opinion was that the standardisation of nurses was eminently to be desired.

### The Colonial Nursing Association.

The ninth annual meeting of the Colonial Nursing Association was held at Sunderland House, by permission of the Duke and Duchess of Marlborough, on Wednesday afternoon last week, Princess Henry of Battenberg, the Patroness, was present, and the chair was taken by the Earl of Westmeath, the President. He referred in terms of high praise to Lord Grey, who had previously occupied his position, and to Mrs. Francis Piggott, Vice-President, who was unable, he hoped only temporarily, to take part in the work of the Association.

Lord Westmeath then drew attention to the steady increase in the number of nurses sent to the Colonies as shown in the report—the number in the year 1897 being six, as against 50 in last year. The number of nurses employed by the Association in the year ending April last was 121, as against 109 in 1903, and of these 94 were in Government service and 27 were private nurses. Having read several letters from nurses describing the hardships they underwent in all parts of the world, and referring to the success of the Cosmopolitan Hospital at Venice, founded by Lady Layard, he stated that he had received a telegram from the Colonial Secretary, who was to have moved the adoption of the report. Mr. Lyttelton regretted that he could not keep his engagement as he found that he had to be at the House of Commons that afternoon.

In his absence, Mr. R. B. Haldane, M.P., moved the adoption of the report. In the course of his remarks, Mr. Haldane said that if we were behind other nations in some things we certainly were not in our scientific knowledge generally, and our medical knowledge in particular, and could not be while we could count among us such men as Kelvin, Lister, and Treves. This Association was the means of sending women to places where they were indeed in a minority, and the managers saw to it that only really qualified nurses were commissioned. Skill, ability, and patriotism were needed, and when women understood that it was the cause of the Empire for which the Association was appealing, they would offer themselves willingly for the work.

Sir Alfred Jones, in seconding the report, said that no one acquainted with tropical countries could fail to recognise what a tremendous movement this was. The London and Liverpool Schools of Tropical Medicine had done much to make matters better for the people in these countries, and in West Africa itself conditions had been greatly improved. There should be qualified nurses in every place; it was a great blessing to a doctor to have a reliable nurse to look after his patients, and there should be institutions where a qualified nurse could train native women to be nurses. Such nurses might not be so good as British nurses, but they would be very helpful. He thought that the association did not sufficiently make its monetary wants known to the public. He believed that they could get more money if they made the matter clear; it was not money wasted, it saved additional expenses incurred by firms who had continually to send men out owing to breakdowns in health. His subscription had been £50 a year, a small one, he admitted, but considerably larger than those of many others who could, he was sure, afford more. He was, however, keenly interested in the work, and to show his interest in a practical manner he would raise his subscription to £250 a year.

Lady Balfour of Burleigh, the President of the Scottish Branch, briefly reported on the work done in the branch. She was followed by Sir John Rodger, who spoke from his own personal experience as governor of the Gold Coast, to the splendid work being done by the Association.

The Chairman then proposed a vote of thanks to Princess Henry of Battenberg for attending the meeting and showing herself really interested as Patroness of the Association. He also expressed the gratitude of the Association to Sir Alfred Jones for his generosity.

A vote of thanks was proposed by Sir Patrick Manson, to the Duke and Duchess of Marlborough for lending Sunderland House, to which the Duchess, in the absence of the Duke, replied, and assured the meeting of the warm interest she and her husband took in the Association.

Mr. Fred Dutton, one of the trustees of the Association, proposed a vote of thanks to Lord Westmeath for having served the Association in so many capacities, and the meeting then terminated.

### Royal British Nurses' Association.

MEETING AND CONVERSAZIONE.

The annual general meeting and conversazione of the Royal British Nurses' Association were held on Wednesday, June 7, in the gardens of the Royal Botanic Society, Regent's Park.

The meeting, which in spite of the inclemency of the weather was attended by about 150 members, took place in the museum of the gardens at 4 p.m.

Dr. Bezly Thorne presided, being supported on the platform by the hon. officers, Mrs. Coster, Mr. Langton, Dr. Godson, and Dr. Berkeley, and the Secretary.

The Hon. Treasurer moved the adoption of the financial report.

This was seconded by Mrs. Latter, who said she thought that Dr. Godson was to be congratulated on its very satisfactory nature. After hearing such a very comprehensive report, it seemed almost superfluous to make any further remark, but she could not help thinking what a very healthy sign the increase in members' subscriptions was. It showed that the Association was not at a standstill but was still growing, and she thought that the members ought to take great credit to themselves for this, and that their success in the past should encourage them to greater efforts in the future. Another point in the report which struck her as being very satisfactory was the increase in the Journal fund. The Journal had always, so far, been a decided loss to the funds of the Association, but this year there was a substantial balance on the right side, and she knew that all the members would join with her in expressing gratitude to their esteemed secretary, to whose untiring efforts the success of the Journal was in a large measure due. Her care and economy in every department of the office was largely the means of placing the funds of the Association on a sounder basis than they had ever been before. In conclusion, she said that it was gratifying to see Mr. Langton still continuing to be interested in, and to work for, the good of the Association.

The Chairman said that although he had been to most of the 16 annual meetings already held, he had never heard so uniformly satisfactory a report. There were one or two items which called for special attention, and although these had already been alluded to, he could not pass them by without a few words. He wished to emphasise the fact that the journal was now able to pay its way, and he felt that they were all very much indebted to the energy, tact and ability of their Secretary. He would also like to say how very much they all appreciated the offices of Dr. Godson as treasurer. He was indeed very warmly to be congratulated on his satisfactory statement, and on the great diligence and interest he had shown in his work for the Association, but, while thanking him for this, they must not forget that the foundation of financial stability had been laid by his predecessor, Mr. Langton. He could not pass over one other item in the report, and that was the handsome donation received from the members in South Africa. He was sure that they all heartily appreciated this evidence of loyalty and good feeling on the part of our sisters in South Africa; it touched his heart to think that they had been so generous in their gifts.

The financial report of the Settlement Fund was then read by Mr. Langton, who moved its adoption.

This was seconded by Miss Tawney, and carried unanimously.

The annual report was then read by the medical honorary secretary, and put to the meeting.

The report stated that various changes have taken place since the last meeting was held. Miss Thorold, the vice-chairman, who felt she could not sympathise with the movement for State registration had resigned, and Dr. Bezley Thorne had been elected to that office. Mr. John Langton finding the work of honorary treasurer too much for him had resigned, and been succeeded by Dr. Clement Godson as honorary treasurer of the General Fund, Mr. Langton having kindly promised to retain the treasurership of the Settlement Fund. Last autumn the President of the Association, Princess Christian of Schleswig-Holstein, visited South Africa. A sure proof of the sympathy the nurses in South Africa have with the Association, and the spirit the Princess has awakened amongst them was made manifest by their donation of £84 to the Benevolent Fund, and further by the number of applications for membership which have been made, so that the opening of a branch out there is being contemplated. Last December the President opened the first of a possible series of settlement homes for the Association's aged members; two members are already installed, and hopes are entertained of two more being added before long.

In seconding the adoption of the report, Miss Forrest said how much they regretted the loss of Miss Thorold's kindly presence, but they felt how difficult her position must have been, objecting, as she did on principle, to one of the main objects of the Association. Of Mr. Langton's services to the Association through long years of stress and strain, she did not think they knew half, or could ever be grateful enough to him for all he had done. With regard to the Journal, the secretary was working single-handed to keep it up to the mark, and it would be a help to her, and make it more interesting to all, if members would let her know of any special local news which would be suitable for publication. Miss Forrest expressed a hope that the lady consuls would see their way this year to arrange for meetings, so as to make each of their districts a real helpful centre of information. She and other members had accomplished this at Bournemouth, and had been amply repaid for the trouble by the interest which it had aroused in others. She would be very pleased to help, by suggestions, any who felt inclined to do likewise.

Dr. Bezly Thorne said that in putting the report to the vote he would like once more to express the great regret felt by every member at the withdrawal of Miss Thorold from office, and to say how very much they all appreciated the great service she had rendered. She had been one of the heartiest and most earnest workers since the very beginning, and, though they had not the pleasure of seeing her with them at the meeting, they wished her long life and health and much happiness, and also that she might soon be converted to their views He thought that the Association ought to be congratulated on having so excellent and devoted a member of the Association to represent it on the Central Midwives Board. He was sure that they were all very grateful to Mrs. Latter. Their thanks were also due to Mrs. Temple Mursell for her services in the Transvaal, and for bringing over the handsome donation from the members in South Africa. The Bill for State Registration had been again introduced before Parliament, and the whole question was now under the consideration of a Select Committee, and had been advocated in a very able manner by several members of the Association.

The General Council was elected for the ensuing year in accordance with the ballot papers sent to all the members, the result of the ballot showing 426 papers signed and unaltered, seven signed and altered, seven unaltered and unsigned, and one altered and signed.

It was proposed by Dr. Godson that Mr. Pitman be re-elected auditor for the ensuing year, and this was seconded by Mrs. Coster who expressed the appreciation of the Association for the good work done by Mr. Pitman for so many years. The proposal was carried unanimously.

Miss East proposed a vote of thanks to the honorary officers for the admirable way in which they had performed their arduous duties, and given so much of their valuable time to the Association.

This was seconded by Mrs. Campbell Thomson, supported by Dr. Bezly Thorne, and carried unanimously.

Dr. Berkeley, on behalf of the honorary officers, thanked the members for their expression of confidence, and said that they were glad to do anything they could for the Association, the more especially as the members were so kind as to be satisfied with their efforts.

Business being completed, tea was daintily served to the nurses and their friends in a marquee erected for the purpose in the grounds, where there was much meeting of old friends, followed by the inevitable comparing of notes and discussion of the meeting and association. Owing to the large number present there was a slight wait for some before procuring tea; this seemed to bring out the spirit of freemasonry which exists among nurses. Crowds are proverbially and, in truth, lonely, but a crowd of nurses off duty seems to be the exception which proves the rule. The grounds were looking lovely after the rain, and proved a haven of delight in contrast to the busy, dirty, dreary streets beyond. The lawns were so fresh and green, the trees just faintly touched with a mystical haze, while some of the pretty costumes worn made a bright touch here and there, completing a charming scene. At the same time the Horticultural Exhibition was taking place in the gardens, so that the conservatories were opened, and in the great palm-house many people were listening to the band of the 2nd Life Guards, which played from three to six. The nurses were able to share this pleasure, and also to enter the flower show, which was most attractive and greatly appreciated, if one can judge by the number of nurses in evidence.

### Opening of the Heywood Johnstone Memorial Home.

On Friday afternoon, the Heywood Johnstone Memorial Home was opened by the Bishop of Southwark. The home is situated in the Old Kent Road, and is intended for persons wishing to be trained as midwives for rural districts. The midwives will attend the cases in the homes of the patients, and will be under the direction of a head and assistant-head midwife. Eight women can be accommodated for training, but at present it is only intended to receive two.

After the Bishop had opened and dedicated the home by prayer, Mrs. Heywood Johnstone, who has founded the home in memory of her husband, who was so largely instrumental in piloting the Midwives Act through Parliament, briefly outlined her aims. As the result of much inquiry, she had found that this particular district was greatly in need of good midwives, and so she had come to the conclusion that by establishing an institution in the locality she would be serving the main end she had in view—the supply of rural districts—and also be helping London's poor. She would like to draw attention to the fact that a charge of 7s. 6d., to be paid in small instalments, was to be made to the patient. It included the necessary attention to the mother and infant for ten days, and provided the doctor when required. By this arrangement she thought that they were avoiding the danger of pauperising the poor and making them fail to realise their responsibility in marrying. There were a few free tickets for extreme cases, and tickets could be bought by anyone particularly interested in a case. The fact that the mothers were to be nursed in their own homes would prove, she hoped, beneficial.

Canon Sinclair, vicar of Cirencester, then spoke a few words, as a personal friend of the late Mr. Heywood Johnstone, and emphasised the interest he took in any work of this kind. He himself, as living in a country town, said that he knew well how much midwives were needed, and he was glad to think that the home would probably send forth some 25 midwives every year. He proposed a vote of thanks to the Bishop for coming to inaugurate the work.

The vote was seconded by Dr. Leary, vicar of St. Philip's, Camberwell.

Colonel Morton, treasurer of the home, having spoken,

Dr. Lionel Smith, who is one of the medical men who have consented to give their names as honorary physicians, referred to the good influence which a nurse might have in a house where she attended during the illness of a mother. He thought that it was an excellent plan to nurse the mother in her own home. A nurse who had had a long training in a hospital does not generally wish to take up maternity work entirely, and, therefore, it was necessary to draw on another class of women with a certain amount of knowledge and plenty of common sense who would obey orders.

The Bishop of Southwark, in replying to the vote of thanks, dwelt at some length on the principles which he hoped would guide the workers in the home. All present would feel that on this occasion it was easy for him to speak. Sometimes it might be difficult to bring in higher thoughts when they were left in the background, but the work of this home was undertaken for the glory of God. The influence of the nurse was very far-reaching. The nurse could be a great power for restraint and could give the patient a glimpse of a better life. He thought the words "He that receiveth you receiveth Me" were helpful on this occasion. It would assist a nurse to realise her responsibility if she felt the true import of these words.

### Great Northern Central Hospital.

FAREWELL TO MISS HULL.

Last week we recorded the presentation to Miss Hull, the retiring matron of the Great Northern Central Hospital, London, from the nursing staff past and present. On Thursday afternoon last week there was another notable ceremony, when those connected with the hospital and other friends assembled to take leave of Miss Hull. The members of the General Committee, the Ladies' Association, the honorary members of the medical staff, and the nursing staff, gathered in the Board-room at 3 o'clock. The chair was taken by Sir John Dickson Poynder, Bart., M.P., chairman of the committee.

Speaking with reference to the circumstance which had brought them together, Sir John referred to the fact that Miss Hull had been 18 years in the service of the hospital; when she first came it was a small, and little-known institution, with only 36 beds, situated in the Caledonian Road. During the period in which she had been associated with the hospital very great developments and changes had taken place in the character of its work, the standard of the nursing and the methods of training generally, and in connection with all these changes her knowledge and experience had been of the greatest value to the Committee, with whom she had always worked in the most loyal and whole-hearted manner. Referring to the high reputation which the hospital held for efficiency and economy and for the excellency of the training of its nurses, the Chairman assured those nurses who were present, that if they followed the example of the matron under whom they had been trained, they could have no better model. He felt how much every one of the Committee, and everyone who had been in any way connected with the hospital, appreciated the work of Miss Hull, her constant and untiring devotion to duty, her unfailing tact and courtesy to all with whom she came in contact, and how deeply everyone felt the parting which was now taking place. On behalf of the Committee, he wished her farewell, and expressed the hope that she might be spared for many years to enjoy a well-earned rest.

Sir John then called upon Mr. Macready, senior surgeon of the hospital, who spoke as the representative of the medical staff and the medical committee in very sympathetic terms of the matron and her work, both in connection with the staff and the patients.

The Hon. Reginald A. Capel said that he was the father of the hospital, having been longer connected with it than any one in the room. He expressed the regard and esteem which he had always personally felt for Miss Hull, and he added that he was certain her example of kindliness and consideration and her influence had been of great value to the nursing staff in connection with their treatment of the patients.

Mr. Fraser Black referred to the fact that he and Mr. Cape were members of the committee who had originally selected Miss Hull for her appointment, and he had been closely connected with the hospital during the 18 years which had elapsed, but he had never at any time or in any case known the matron to do anything but the right thing, and what was expected of her. He then presented her on behalf of the members of the committee and the medical staff, and of some of the retired residents, with a handsome diamond brooch, which, as he put it, was "a diamond brooch for a diamond matron!"

Sir John Dickson Poynder, on behalf of the Ladies' Association of the hospital, then presented Miss Hull with a gold watch and chain and a tea basket, the watch being inscribed inside the case with the words:—"From the Ladies' Association of the Great Northern Hospital, 1905." An illuminated address was also presented by the members of the committee.

Mr. Harry Bevoir, on behalf of Miss Hull, expressed her thanks and acknowledgment for all that had been said, for the handsome gifts which she had received, and yet more for the loyal support which she had always enjoyed and the kindness she had experienced from the members of the staff.

Miss Hull sailed on Saturday from Southampton for South Africa, whence she will proceed to Australia, China, and India visiting friends and relatives.

### London Homœopathic Hospital.

### FÊTE AND BAZAAR.

Most successful results attended the efforts of the Ladies' Guild of the Homœopathic Hospital, under whose auspices a garden party and sale of work was held for the purpose of financially assisting the Institution, at 11 Kensington Palace Gardens, the residence of Mr. R. W. Perks, M.P., on Thursday last week. The Countess Cawdor, President of the Ladies' Guild, who was accompanied by her two daughters, opened the proceedings at 3 o'clock, and wished the fête every success. Mr. J. P. Stillwell, J.P., Chairman of the Hospital's Board of Management, returned thanks to Lady Cawdor, and Lord Calthorpe having replied on her behalf, the business of the day commenced.

On the lawns, where tea was served, the Aëolian Ladies' Band, under the baton of Miss R. Watson, contributed largely to the pleasure of a very appreciative audience. Particular interest attached to the Fine Art stall, presided over by Mrs. W. Marchant, which not only exhibited many pictures, etchings, etc., of well-known artists, but was also displaying the drawing "A Messenger of Love," drawn especially for the Bazaar by Sir L. Alma-Tadema, R.A.

The dramatic entertainments and concerts, of which both professional artistes and amateurs were exponents, proved deservedly popular features of the fête. It was intended to have one particular nurses' feature in the bazaar, but owing to lack of space this project was abandoned. Several nurses, however, were present from the hospital, many of them flitting about in full uniform gave assistance in various ways. Several members of the honorary medical staff of the hospital were also present at the opening ceremony, besides many other persons of rank who had granted their patronage.

Late in the evening the result of the fête was made known, and the Council of the Ladies' Guild handed over to the hospital the sum of £1,000. Of this amount £192 10s. came from the purses of £5 and upwards collected by children and presented by them to Lady Cawdor.

### Appointments.

**[No charge is made for announcements under this head, and we are always glad to receive and publish appointments. The information, to insure accuracy, should be sent from the nurses themselves, and we cannot undertake to correct official announcements which may happen to be inaccurate. It is essential that in all cases the school of training should be given.]**

Bridgend Cottage Hospital.—Miss Mary Marshall has been appointed matron. She was trained at the Monsall Fever Hospital, Manchester, and the Royal Devon and Exeter Hospital. She has since been sister at the Eston Hospital, Yorkshire.

Cumberland Infirmary, Carlisle.—Miss Kate Baker has been appointed night sister. She was trained at Bristol Royal Infirmary, where she has since been sister of the gynæcological and ophthalmic wards.

Lichfield Workhouse Infirmary.—Miss Edith S. Butler has been appointed superintendent-nurse. She was trained at Prescot Union Infirmary and St. Helen's Fever Hospital. She has since done private nursing at Oldham, and has been ward sister at the Union Infirmary, Fir Vale, Sheffield. She is registered under the Central Midwives Board.

Southwark Union Infirmary.—Miss Margaret Aisthorpe has been appointed ward sister. She was trained at St. George's Infirmary, Fulham, and Southend Borough Fever Hospital. She has since been staff nurse at the Miller Hospital, Greenwich.

The Federated Malay States.—Mrs. C. A. Warren has been appointed nurse in the Federated Malay States. She was trained at Guy's Hospital, London, received midwifery training at Edinburgh, was sister in the Burgher Camps, Transvaal, and sister in the Plague Segregation Hospital during the epidemic at Durban, 1903.

Victoria Hospital, Keighley.—Miss Margaret Storey has been appointed matron. She was trained at Leeds General Infirmary, and has since been matron at Fleetwood Cottage Hospital, Palmer Memorial Hospital, Jarrow, and Newtown Infirmary.

Wimbledon Cottage Hospital.—Miss Lilian D. Field has been appointed matron. She was trained at University College, Hospital, London, and has since held posts at the Salop Infirmary, Shrewsbury, Hull Royal Infirmary, and the Hospital for Women, Soho Square, London.

### A Book and its Story.

### THE STORY OF FIVE YEARS IN THE NEW HEBRIDES.*

Dr. Lamb in "Saints and Savages" recalls the experiences of a five years' sojourn in the New Hebrides as a Presbyterian medical missionary. These experiences he has put together in more or less narrative form, with the result that a very interesting book has been made, for, as he explains, the tapestry of the story has been woven on the loom of actual life, a life where the forces, good and bad, now at work, are fast sweeping away every vestige of the old order of things. A glimpse of the heathen is given as they were seen by the writer—a glimpse which enables us to understand in a measure "how they lived and what they lived for, before white sails were first seen flitting among these islands, bringing a disturbing element into their simple lives." "These islands are a gem among the possessions of Australia. The story of Eden is being repeated. The black man has been tempted to eat of the tree of knowledge. The result is the same—death to his race and expulsion from his fair heritage."

"Saints and Savages" represents a study in black and white; the contrasts are strongly marked, as the title indicates, but no attempt has been made by the writer to define the terms, saint and savage. He adds: "In our young days it seemed an easy matter; easy also on paper, but contact with the real men and women alters all that. And such are these (the natives). The gradations as we pass from one extreme to the other are so fine, that we may say with the ancient Rabbis, 'Between Gehenna and Paradise there is only the breadth of two fingers,' Probably the originator of that saying meant the chink between the fingers. Nevertheless, the chink is a fact and represents the line of cleavage."

When cruising round the northern islands in search of a healthy site for a central hospital to serve the whole group there were two that had particular claims to attention. Santo, in the north-east, and Ambrym. Santo is the largest in the group, and it is the first extensive one that was discovered in the Pacific. But it was too far from the centre to serve the purpose, and for other reasons the choice fell on Ambrym.

Ambrym is described as being a garden of cocoa-nut palms, and for its size the most populous island of the group. Selwyn, Patteson, Copeland, Mackenzie, Paton, and others had landed there, and two predecessors of Dr. Lamb had built a house in the north-east corner of it. They, after a short term of arduous service, had broken down in health, and been compelled to retire. The island had for a few years been without a missionary, and the committee in New Zealand were anxious that Dr. Lamb should set to work for them again. Ambrym was later the base of his labours.

Some description of the New Hebrides and its sister groups of islands may be interesting here to those of our readers who may be strangers to their history.

"The New Hebrides and sister groups form a strange chapter in the study of human history. Here life is at its lowest ebb, and the people are as the washed-up foam and *débris* at the margin of sand and wave. This group forms a line of demarcation between East and West. Tribes have come from various directions, and have fought for and obtained a footing. By tribes we mean districts that speak the same tongue. Organised tribes there are none. In origin the children of Ham, they came from Asia. Their footprints have been traced through India and down the Malay Peninsula to the Pacific. As centuries passed they were driven eastward—as the Celts were to the west—by stronger tribes and new invasions, onward and forward, till, about 700 years ago, the first canoes are believed to have reached the islands."

Of the beliefs of the islanders Dr. Lamb writes as follows:—"To them the whole world is limited to the visible horizon, and their own island is, of course, the hub of the universe. The creation of the world they attribute, in Ambrym, to an old man who lived long ago, Parakulkul. Their religion is concerned with an order of invisible beings. Spirits or ghosts are supposed to exercise their powers through curiously-shaped stones, to which remarkable powers are attributed. The power of making the cocoa trees flourish, the yams to grow large, and the pigs to multiply, among other things."

A photograph is given showing "a spirit-stone" obtained by an Englishman, Mr. Mansfield, from the son of a chief. It is hard and brittle and apparently of volcanic origin. Yet it is very heavy and unlike the pumice-stone seen on Ambrym. The natives are thought by ethnologists to have been on these islands since A.D. 1200, and as the stone has been handed down from generation to generation, it may possibly have been brought hither by those who came first. . . . The man who first found the stone must have observed its weirdly human appearance, and by a little polishing and carving have increased the likeness. The natives have always believed in the existence of two great spirits whom they call the Spirit of Light and the Spirit of Darkness. White men they call by the same name—Vyu—the Spirit of Light—because they think they have come down from the sky. The creator of the world, Parakulkul, is the spirit of a dead ancestor and great chief. At one time men were four-footed animals, and walked on all fours—that is all men walked upright from the beginning, but the lazy ones who would not work came to walk on all fours and degenerated into animals like pigs and cows and goats, and had faces like them. When men die they go down into a world of spirits. When a chief dies he goes down with all his bravery of ornament; but it is only the shadow or appearance of these things that are on him. The world below is like this world, only it is shadowy and ghostly. There the spirits of men live always. They do not worship or pray to anything. When in trouble they call on the spirit of their father or friend, and that spirit may come from the lower world to help them. They do not sacrifice to anything. But at the feast of the dead a man's image is painted afresh, and pigs are killed before it out of respect to his memory, and to show he is not forgotten. Many other beliefs and practices are spoken of, but their doctrine of rewards and punishments is as follows. They count generosity as the highest virtue, and meanness as the lowest failing in a man's character. To the generous man is reserved a place in 'the good place,' and he will be saved. To the mean man a future in which he will be compelled to wander as an outcast pig, and as miserable and malignant wanderer, he may come back to earth and haunt the graves and woods and do evil to men. To those who die young a happy future among the pleasantest place of all is allotted. The village is bright, flowers abound, and there is much singing and dancing.

This information was given by a native, who said: "The women think these things, and they grieve over those who die young—the mothers most of all—and they esteem most those who are shot for them, who pay the price of death and die on their account." "Saints and Savages" clearly proves that the effect of missionary labour is to make saints of savages in spite of all that is said, so often, to the contrary.

* "Saints and Savages." By Robert Lamb, M.A., M.B., etc. (W. Blackwood and Sons. 6s.)

### Everybody's Opinion.

### THE GENTLE CLASS.

"A Professional Man's Daughter" writes: I am not surprised at your representative having misunderstood Miss Shannon, when, in giving her evidence before the Select Committee of the House of Commons, she did not class professional men's daughters as being gentlefolk. Considering that two of the members of the select committee are professional men, let us hope that they, too, misunderstood her.

### A RULE AND ITS CONSEQUENCES.

"Matron writes: As your correspondent, "One of the Staff," considers the committee of the Stockton-on-Tees Nursing Association hard in having asked their matron to resign, I wonder if my case will help her to see that other committees can be unjust as well as hard. Improbable as I know it sounds I was ordered to resign for no other reason than that the doctor did not come when I sent for him, though he admits receiving my note an hour and a-half after it was delivered at his house. I have worked here fourteen years, and judging from the expressions of regret and tokens of gratitude I have received, the patients must have appreciated the attention they had while under my care. Unfortunately for us who are of middle age, the committees and staffs who appointed us and appreciated our efforts to do our duty are gone, and the new members wish for new brooms. I was told two years ago that ways and means were being discussed for getting rid of me, the great obstacle being that I am "a very good nurse." Having to earn my living, I was thankful that I had one redeeming quality. Putting it down to servants' gossip, if possible, I devoted myself closer to my duty, and I have always tried to obey every wish of the committee.

### IN DEFENCE OF PRIVATE NURSES.

"Justice" writes: So much is talked about at the present time of the higher education of women, State registration, etc., that a few words in the private nurse's defence will not come amiss. I am a private nurse, and I find that a great many people are prejudiced, not unnaturally, against private nurses because of what they may have heard their friends or relations say about some unfortunate experience they may have had with a private nurse. My experience has been that a great many of my nurse-friends have had "words" and left cases because instead of the householders getting it into their heads that the nurse came to look after her patient only, they think she ought to help in the general household duties, such as shopping, cooking, not for the patient, but for the family, as well as other various duties which fall to the lot of a maid and not a nurse. I do not say that all people are alike in their treatment of nurses. I have been exceedingly well treated in all my cases except one, and it is this one that has aroused my indignation. No true woman would refuse to put her hand forward in an emergency, but day in and day out for a private nurse to be asked to do general household duties is, in my opinion, very far from right. I daresay that some private nurses have had a similar experience to mine and can therefore fully appreciate my statements. My contention is that if we nurses go through three years' hard training and study hard to make ourselves perfect in the profession we are not to be put on a level with servants and be expected to do their work as well as our own, even if the case should be a light one? I should much like to hear what other nurses have to say on this matter.

### THE NEED FOR REGISTRATION OF NURSING HOMES.

"A Victim" writes: Reading with interest letters in The Hospital on "Money-making Institutions," and being one who has suffered through joining one of these private adventure co-operations (so called), I should like to give my experience. I am, by the way, a three years' certificated nurse, recently trained in a large hospital. Upon leaving my training school I joined a private adventure co-operation, without a committee, in a large city in Lancashire. I then discovered that the nurses paid 4s. a week (all the year round, whether at a case or not, and always in advance) as commission to the institution; also, when not at work they lived in the home, and paid a further 10s. per week for their board. I remained on this staff 12 months, and during all that time had two cases given to me by the Principal of the institution. One lasted eight weeks and the other two weeks. The fee weekly was one guinea and a half, and I earned altogether £15 15s. Out of this I paid £10 8s. in commission to the institution, leaving £5 7s. towards my board and all other expenses, such as uniform, clothes, and laundry, etc., for nearly 10 months. Fortunately, I had friends in the neighbourhood and was well known. My friends helped to provide me with cases, quite independent of the Institution. I was, however, still expected to pay the commission of 4s. weekly to the Principal, although I took the work through friends—and, even with my friends' help, there were sometimes intervals of five and six weeks during which I had no work, and had to board in the Home. I was by no means the only nurse on that staff who was out of pocket. There are several of these so-called co-operations in the city to which I refer, and doubtless some in other large towns. They often secure large staffs of nurses—25 or 30, and even more—though not by any means always fully-trained and certificated. They are under no committee, and can consequently make what rules they please to their own advantage. I leave it to be imagined, if my experience was so unfortunate with friends to render aid, what would be the plight of a nurse joining that staff from a long distance, knowing no one in the place, having to depend absolutely on work given to her through the Principal of the Co-operation? Some may think, no doubt, that a nurse can always, on finding things unsatisfactory, resign her post on such a staff upon giving one month's notice, or paying one month's commission in lieu of notice. But it is not always easy, or even possible, to get on to another staff without delay. Often the change means months of waiting for a vacancy, and it is equally difficult sometimes for a nurse to obtain work on her own account. Surely some form of registration and inspection making it impossible for these so-called Co-operations to continue on their present basis, would be welcome to nurses.

### Death in Our Ranks.

We regret to record the death of Nurse Mary Jones, who died suddenly on Thursday, June 8th, at her post as charge nurse at Church Hill House Open-Air Sanatorium, Ventnor. She was trained at Boston Hospital.

### TRAVEL NOTES AND QUERIES.

By our Travel Correspondent.

A Fortnight in Belgium for Four People (Kent).—I do not think children ever enjoy the Continent very much, but if you must take them you can manage the following plan on the sum you mention:—Second class return to Ostend from Tower Bridge 9s. each. From there take train to Bruges near by, and then steam tram to Knokke, which being by the sea will please the children. Go to Hôtel de Bruges and ask for rooms on the fourth floor. If it is full go to Hôtel de la Marine, both are cheap. When you have spent a week there go to Bruges and stay at the Hôtel Panier d'Or. There is much to see there. If funds hold out you might make a day's excursion to Courtrai and Oudenarde, but leave the young ones behind, they would not care for such an outing.

Ostend for a Fortnight (Jour de Fête).—Ostend is an expensive place, and the sum at your disposal is small for the length of your holiday, but with care it can be managed. You cannot manage for yourselves, being strangers to the country and language, go to Hôtel and Café Royal in the Rue de la Chapelle. I think Mr. Schmid, the proprietor, will take you for 7 frcs. per day on the fourth floor. This would come roughly to £8 for both of you for the fortnight. Go from Tower Bridge by G.S.N.C. Return fare, second class, 9s. When you have paid all tips your £10 will be gone, but out of your reserve fund you can visit in day excursions, Bruges, Courtrai, Oudenarde, and perhaps Ghent.

### Notes and Queries.

**REGULATIONS.**

**The Editor is always willing to answer in this column, without any fee, all reasonable questions, as soon as possible.**

**But the following rules must be carefully observed.**

1. **Every communication must be accompanied by the name and address of the writer.**
2. **The question must always bear upon nursing, directly or indirectly.**

**If an answer is required by letter a fee of half-a-crown must be enclosed with the note containing the inquiry.**

*Rural District.*

(89) Will you kindly tell me whether I can accept a post as district midwife and cottage nurse combined in a rural district?—*A. L. K.*

At present such an arrangement is feasible.

*Registration of Midwives.*

(90) I wish to have my name placed on the register for midwives. Would you be kind enough to let me know to whom to apply for all particulars?—*Nurse C.*

Write to the Secretary of the Central Midwives Board, 6 Suffolk Street, Pall Mall, S.W.

*Peptonised Milk.*

(91) Is it necessary to boil peptonised milk when taken by mouth as it is when taken by the rectum?—*Nurse M.*

The usual way of peptonising milk to be taken by the mouth is by adding a Fairchild Zymine Peptonising Powder to one pint of milk (diluted with a quarter of a pint of water), keep warm 20 minutes, boil and sweeten.

*Physical Training.*

(92) Is there a physical training college at Bedford where they train students in medical gymnastics, massage, and other branches of this kind?—*M. T.*

Write to Miss Margaret Stansfeld, 37 Lansdowne Road, Bedford, or to the Hon. Sec., British College of Physical Education, 7 Johnson Street, Notting Hill Gate, London, W.

*Probationer.*

(93) There was an advertisement in THE HOSPITAL from the ——— Hospital, for probationers. I have made inquiry and had a paper from them, but cannot tell what kind of hospital it is. I have procured "The Nursing Profession: How and Where to Train" but cannot find the hospital mentioned. . . .—*N. W. A.*

The hospital is mentioned in "Burdett's Hospitals and Charities" as having 80 beds, and a resident staff of two house surgeons, a matron, and 21 nurses. You would be wiser, if you are thinking of taking up nursing professionally, not to train in any hospital containing less than a hundred beds.

*Royal British Nurses' Association.*

(94) Will you kindly tell me where to write to get particulars about joining the Royal British Nurses' Association?—*L. F.*

Write to the Hon. Secretary, 10 Orchard Street, London, W.

*Cottage Hospital.*

(95) I should be obliged if you would tell me what you think the best department of nursing to take up? I am a three years' certificated hospital nurse, have been private nursing for three years and have got on very well indeed, but find now that my mother, who is a widow and quite alone, needs me more at home. I can speak Spanish and understand French, but would rather get something to do near here. I had thought of going out to cases by the hour or visit, but there is not scope for that in this town. Do you think it would be possible to get a post as matron of a cottage hospital where they would allow mother to live as well?—*Scotland.*

It might be quite possible for you to arrange to keep your mother with you if you were a district nurse.

*Elderly Nurses.*

(96) Can you tell me if there is a society for elderly nurses to help them to obtain suitable posts?—*M. T. N.*

Write to the Hon. Secretary, Royal British Nurses' Association, 10 Orchard Street, W.

**Handbooks for Nurses.**

| | Post Free. |
|---|---|
| "How to Become a Nurse: How and Where to Train." | 2s. 4d. |
| "Nursing: its Theory and Practice." (Lewis.) ... | 3s. 6d. |
| "Nurses' Pronouncing Dictionary of Medical Terms." ... | 2s. 0d. |
| "Complete Handbook of Midwifery." (Watson.) ... | 6s. 4d. |
| "Preparation for Operation in Private Houses." ... | 0s. 6d. |

Of all booksellers or of the Scientific Press, Limited, 28 & 29 **Southampton Street, Strand, London, W.C.**

### For Reading to the Sick.

COMPENSATIONS.

I SORROWED that the golden day was dead,
Its light no more the country-side adorning;
But whilst I grieved, behold the east grew red
With Morning.

I sighed that merry Spring was forced to go,
And doff the wreaths that did so well become her;
But whilst I murmured at her absence—lo
'Twas Summer.

I mourned because the daffodils were killed
By burning skies that scorched my early posies;
But while for these I pined, my hands were filled
With roses.

Half broken-hearted I bewailed the end
Of friendship, than which none had once seemed nearer;
But whilst I wept I found a closer friend
And dearer.

Thus I learned old pleasures are estranged,
Only that something better may be given—
Until, at last, we find this earth exchanged
For Heaven.

*Anon.*

We all want quiet; we all want beauty for the refreshment of our souls. Sometimes we think of it as a luxury, but when God made the world, He made it very beautiful, and meant that we should live amongst its beauties, and that they should speak peace to us in our daily lives.

*Octavia Hill.*

I have noticed that wherever there has been a faithful following of the Lord in a consecrated soul, several things have inevitably followed, sooner or later. Meekness and quietness of spirit become in time the characteristics of the daily life. A submissive acceptance of the will of God, as it comes in the hourly events of each day; pliability in the hands of God to do or to suffer all the good pleasure of his will; sweetness under provocation; calmness in the midst of turmoil and bustle; yieldingness to the wishes of others, and an insensibility to slights and affronts; absence of worry or anxiety; deliverance from care or fear—all these, and many similar graces, are invariably found to be the natural outward development of that inward life which is hid with Christ in God.—*H. W. S.*

But if meantime we ever think, in the anguish of some great affliction, or the weariness of some night of sorrow, that because He is thus dealing with us He cares not for these our sufferings, that He looks but to the end, and takes no note of the bleeding feet or the burning brow, or the sickness of the heart of those whom He is leading, how greatly we shall wrong his love. Surely they who have longest been journeying under His guidance will most readily testify that this is not so. They know something of the sympathy of Him who is very Man, who bore more than He asks us to bear, who is evermore touched with a feeling of our infirmities, who wept with Martha and Mary at the grave of Lazarus, who sends no sorrow but He sends with it a healing balm, nay, who comes Himself to His afflicted to breathe strength and peace into their hearts.—*Rev. T. V. Fosberry.*

